# Real-world car-to-pedestrian-crash data from an urban centre

**DOI:** 10.1186/1752-2897-4-2

**Published:** 2010-02-16

**Authors:** Uli Schmucker, Melissa Beirau, Matthias Frank, Dirk Stengel, Gerrit Matthes, Axel Ekkernkamp, Julia Seifert

**Affiliations:** 1 Dept of Orthopedic and Trauma Surgery, Unfallkrankenhaus Berlin, Warener Strasse 7, 12683 Berlin, Germany; 2 Dept of Orthopedic and Trauma Surgery, Ernst-Moritz-Arndt-University Greifswald, Sauerbruchstrasse, 17475 Greifswald, Germany

## Abstract

**Background:**

Pedestrians are at a high risk for crash and injury. This study aims at comparing data from real world crashes with data gathered from experimental settings.

**Methods:**

IMPAIR (In-Depth Medical Pedestrian Accident Investigation and Reconstruction) was a prospective, observational study performed in a metropolitan area. Data was collected on-scene, from clinical records, and interviews. Data comprise crash data, details on injury pattern and injury severity.

**Results:**

Thirty-seven pedestrians (of which 19 males) with a mean 37.1 years of age were included in the study. The mean collision speed was 49.5 km/h (SD 13.7, range, 28 - 93). The mean ISS (31.0, SD 25.4) and the 24% fatality rate indicate a substantial trauma load. The most common AIS 4+ injuries were to the head (23 subjects), followed by chest (8), pelvis (4), and abdomen (2). An association of impact side and injury side (right/left) was found for abdominal, chest, pelvic, and upper limb injuries. Primary head impacts were documented on the windscreen (19 subjects), hood (10), A-pillar (2), and edge of the car roof (2). With bivariate analysis, a significant increase of MAIS 4+ head injury risk was found for collision speeds of >40 km/h (OR 9.00, 95% CI 1.96-41.36).

**Conclusion:**

The real-world data from this study is in agreement with previous findings from biomechanical models and other simulations. This data suggest that there may be reason to include further pedestrian regulations in EuroNCAP.

## Background

In 2008, 695 pedestrians have been killed and 33 733 injured in Germany [1] as consequence of a road traffic crash (RTC). Previous research reported a high crash and injury risk for pedestrians [2-5]. Pedestrian crashes were found to be associated with high injury severity and mortality when compared to other road users [2,3,6-13].

The significant impact of vehicle design on injuries of pedestrians is undoubted [2,11,12,14-17]. Manufacturers recognized safety engineering as an important marketing strategy. In this context, EuroNCAP, a consortium of European agencies, has established test protocols to inform consumers about the experimental safety performance of individual car models in different impact scenarios including pedestrian collisions [18]. However, the external validity of findings gathered under laboratory conditions is unclear, and remains an ongoing issue of debate.

In-depth analysis of traffic crashes from both a technical and a biomedical perspective may overcome the limits of simplified, artificial experimental settings. In this study, a team of technicians and physicians collected crash and injury of urban pedestrian crashes. We aimed at comparing experimental data with data gathered from real-world collisions.

## Methods

### Study design

The IMPAIR study (In-Depth Medical Pedestrian Accident Investigation and Reconstruction) was a prospective, observational study planned to supplement the available evidence from crash tests by reliable data from urban road traffic casualties. The study was approved by the Institutional Review Board, and all subjects provided written informed consent. In case of altered consciousness, death, and under aged subjects, written informed consent was sought by legal representatives. Initial data recording on scene was permitted by the district's public prosecutors department.

Between July and December 2004, consecutive pedestrian crashes occurring in a predefined urban area in Berlin, capitol of Germany, were enrolled in the study. This report is confined to cases with complete technical and medical logs. To create a homogenous sample, only car-to-pedestrian collisions involving vehicles registered after 1995 were included. Vehicles were categorized as 'cars' according to federal licensing regulations. No van, sports utility vehicle, truck, lorry, motorized two-wheeled vehicle, etc. was included. Eligible subjects had sustained an injury as a pedestrian due to collisions with the cars front at a minimum collision speed of 20 km/h (appr. 13 mph). We excluded pedestrians on roller skates, skateboards, inline skates, etc., handicapped pedestrians (e.g. using crutches), and rollover subjects.

### Data collection

Field teams consisted of one technician and physician each. Calls were received from a central dispatcher in duty for medical emergencies. The technical staff comprised independent engineers from DEKRA http://www.dekra.com, an international service provider for advanced automotive and traffic safety. Data collection on the spot, including photo documentation, comprised crash environment, separated vehicle parts, braking tracks, body contact points, extent and location of deformation. Evaluation of crash and impact characteristics was performed by engineers and physicians. Data on injuries comprises detailed description of every injury irrespective of location or severity. Although the study was conducted in 2001-2004, we re-coded injury data according to (Maximum) Abbreviated Injury Scale 2005 ((M)AIS)[19] and Injury Severity Score (ISS) [20]. Survivors were examined and interviewed during their hospital stay, and data from hospital charts were added. In fatalities, injuries were traced from autopsy reports.

Data are presented as means, medians, standard deviations (SD), and proportions, according to the underlying distribution. Odds ratio was calculated with bivariate analysis for assessment of impact characteristics and resulting injuries. The independent samples t-test was used for comparison of mean values. Where suitable, 95% confidence intervals for central estimates, differences in means and proportions, or ratios were calculated. Statistical significance was set at p = 0.05. The Stata 8.0 software package (Microsoft, Vermont, USA) was employed for all analyses.

## Results

160 road traffic crashes involving pedestrians were recorded, of which 37 (23%) fulfilled entry criteria. Those 123 subjects not available for the study comprise 2 skateboarders, 3 inline skaters, 3 pedestrians using crutches, and 23 refusing written consent. For 17 subjects, essential clinical information was missing. For 56 subjects, technical on-scene information was incomplete. The latter mainly resulted from immediate clearance of the scene by police forces. The study sample comprised 19 men and 18 women with a mean age of 37.1 years (SD 23.8), including 11 under aged subjects (range 8-17 years). All except one collision occurred on roads with speed limits of 30 or 50 km/h. The mean collision speed (49.5 km/h, SD 13.7 km/h, range 28 - 93 km/h) was higher (55.1 versus 43.0 km/h) in those 18 collisions without braking in the pre-crash phase (p = 0.006).

### Injury pattern

A mean Injury Severity Score ISS 31.0 (SD 24.4, range 3-75) was documented. Table 1 presents injury pattern and severity by case. Nine subjects died (mortality 24%), of which 3 died on the spot (on-scene mortality 8%). Twenty-three subjects (62%) sustained at least one MAIS 4+ injury, all of which were to the head (23 subjects), or in addition to the chest (8), spine (6), pelvis (4), or abdomen (3). Presence of an MAIS 4+ head injury was found to significantly increase the risk of fatal outcome (OR 1.64, 95%CI 1.18-2.28) and an additional MAIS 4+ injury to a second body region (OR 1.92, 95%CI 1.30-2.84). Significantly more MAIS 2+ injuries sustained were to the head, neck, or face (p < 0.001) than to any other body region. MAIS 2 + injuries including the head and lower limb was the most frequent pattern in multiple injured subjects (21, 57%, p < 0.001).

**Table 1:** Injury pattern and severity by case

	**MAXIMUM ABBREVIATED INJURY SCALE (MAIS) **[19]					
	
ISS*	head, neck	face	chest	abdomen, pelvic contents	upper limb	lower limb, pelvic girdle
3	1	1	0	0	1	1
3	0	1	0	1	1	1
6	1	1	0	0	2	2
6	2	1	1	0	1	1
6	1	0	0	1	1	1
6	1	1	0	0	2	2
6	2	0	1	1	1	1
9	2	1	0	0	2	2
9	2	2	0	0	1	1
14	2	0	0	0	1	3
14	1	1	3	0	2	1
14	3	0	0	0	0	2
17	2	1	0	2	2	3
18	4	1	0	0	1	1
18	4	0	0	0	1	1
21	4	2	0	0	1	1
21	4	1	0	0	1	2
22	3	1	3	1	1	2
24	4	2	1	1	1	2
24	4	2	0	0	1	2
26	4	1	0	0	1	3
26	4	1	0	0	3	1
29	4	0	3	0	2	2
30	5	1	0	0	2	1
33	5	1	0	2	1	2
35	5	1	0	0	1	3
43	5	1	3	3	1	1
50	4	1	5	3	1	3
50	5	2	4	0	2	3
55	5	1	4	0	2	2
59	5	1	3	5	1	2
75	4	0	6	3	1	3
75	5	2	4	6	2	1
75	4	1	5	6	2	2
75	6	1	5	1	1	2
75	6	1	5	3	2	2
75	6	1	3	0	1	2

MAIS group	NUMBERS OF REGION-SPECIFIC INJURY n (% of 37 subjects)					

0,1	6 (16)	31 (84)	23 (62)	28 (76)	24 (65)	14 (38)
2,3	8 (22)	6 (16)	6 (16)	6 (16)	13 (35)	23 (62)
4,5	20 (54)	0	7 (19)	1 (3)	0	0
6	3 (8)	0	1 (3)	2 (5)	0	0

* Injury Severity Score [20]

Most common sustained injuries to the head were subarachnoideal bleeding (17 subjects) and brain contusion (14). With regard to chest injuries, serial rib fractures (7) and lung contusions (7) were most frequent, while spleen (5) and liver rupture (5) were the most frequent abdominal injuries. Unstable thoracic (4) and cervical spine fractures (3) were the leading spine injury, while anterior pelvic ring fractures (5, of which 3 had additional sacral fractures) were documented as leading pelvic injury. Five fractures of the humerus, 8 closed and 7 open tibial/fibular fractures, and 8 complex knee joint instabilities were documented as most common sustained limb injuries.

### Impact characteristics and resulting injuries

Collision speed was significantly higher in fatalities when compared to survivors (61.9 and 45.1 km/h respectively, p < 0.001). Except for one case, collision speed was >50 km/h among the fatalities (range 39-93 km/h), while the survivors crashed at 28-65 km/h. Among the 37 subjects, 21 were primarily impacted on the left body side and 16 on the right body side. Table 2 illustrates the distribution of match (vehicle impact and injury on same side), mismatch (on opposite side), and of those injuries that were documented either bilateral, multiple, or diffuse (e.g. diffuse axonal injury). In head injuries, no clear dominance of selected injuries or injured areas was found. Of the 24 seriously head injured, 23 showed multiple marks in different regions of the head or face. Regarding chest, abdominal, and pelvic injuries, both matches and multiple/bilateral injuries are frequent. Regarding the upper extremity, all MAIS 2+ injuries occurred on the vehicle's impact side except for one on the opposite side and two bilateral injuries. MAIS 2+ injuries of the lower limb stand out for a high proportion of bilateral injuries, while unilateral injuries were mostly found on the vehicle's impact side.

**Table 2:** Distribution of vehicle impact to pedestrian by injured per body region

INJURED REGION, MAIS 2+, n			
	multiple* n	match* n	mismatch* n
Head, face	17	8	6
Chest	6	6	2
Abdomen	4	3	1
Pelvis	5	7	2
Upper limb	2	10	1
Lower limb	11	10	2

*multiple is valid for injuries not located in a particular region, thus bilateral or diffuse, e.g. bilateral limb fractures, diffuse axonal injury match is valid if injury is on the same side as primary vehicle impact (left or right), mismatch is valid if injury is on the opposite side

### Head impacts and injury characteristics

The mean collision speed was significantly higher in fatal outcomes, in ISS > 15, and in MAIS 2+ injuries of the head, chest, spine, abdomen, and lower limb (all p < 0.001).

**Figure 1:**
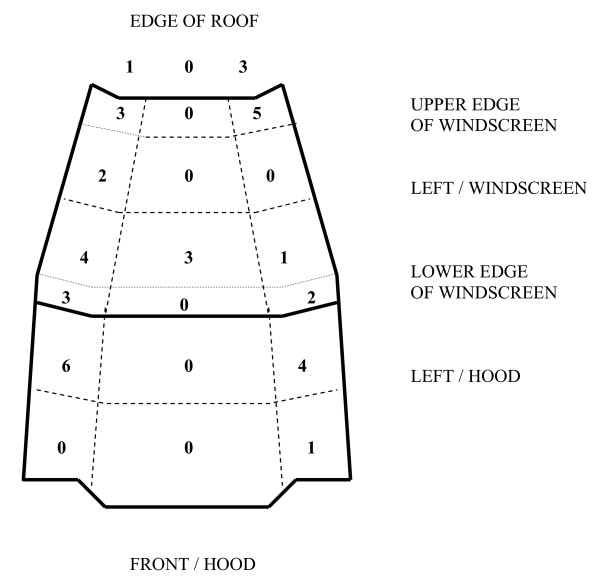
**Location of primary or secondary head impact on hood, windscreen, and edge of roof**.

Figure 1 illustrates the distribution of head impacts. The first head impact was found on the windscreen (19 subjects; 51%), hood (10; 27%), road surface (3; 14%), A-pillar (2; 5%), or edge of the car roof (2; 5%). A second head impact was documented in 8 subjects, which were to the windscreen (4), roof edge (2), road surface (1), or hood (1). One subject sustained no head impact. In those 23 subjects with primary or secondary head impact to the windscreen, the impact either occurred to the upper third (10 subjects) or the lower third (13) of the windscreen. Thirteen subjects impacted close (0-10 cm) to the rigid upper or lower edge of the windscreen or the A-pillar (2) (Figure 1). This increased the risk for MAIS 4+ head injury though not significantly (OR 4.4 95% CI 0.8-25.2). On the other hand side there was a significantly lower crash speed (46.1 versus 56.0 km/h, p = 0.04) in those subjects who did not impact the rigid frame of the windscreen. 4/23 head impacts included an impact to the wiper. Among the 11 head impacts to the hood, 10 occurred to the posterior half and 1 to the frontal half of the hood. The risk of a MAIS 4+ head injury increased though not significantly in impacts to the windscreen, roof edge, or A-pillar when compared to impact to the bonnet (OR 1.89, 95% CI 0.39-9.09). However, the documented crash speed was significantly lower in the latter group (43.1 versus 53.4 km/h, p = 0.05). A significant increase of MAIS 4+ head injury risk was found for collision speed >40 km/h (OR 9.00, 95% CI 1.96-41.36).

A head injury due to impact on the road surface was documented in 4 subjects, of which 3 had no head impact to the vehicle at all. The latter sustained only minor (AIS 1) head injuries while the remaining subject (with primary head contact to the vehicle and second to the road surface) suffered from an MAIS 5 head injury.

## Discussion

This study provides detailed technical and medical information on car-to-pedestrian-collisions in a well defined sample, and adds real-world data to previous experiments. However, limits of this investigation merit discussion. Only one quarter of all documented cases were included in the analysis. The main reason was missing technical information because of cleaning of scene by police forces. In consequence, the study sample is small which limits the meaningfulness of the principal study findings. On the other hand side, each case provides detailed crash and injury data which is a strength of this study.

Car-to-pedestrian crashes proved to be a significant source of morbidity and mortality. The observed mean ISS of 31.0 is at the upper limit compared to other published series (ISS range 9-34 [7,9,10]). This may result from exclusion of cases with a collision speed of < 20 km/h.

Our results are consistent with previous investigations which identified head injuries as leading cause of death [7,10,11,14,21,22]. In contrast to a post mortem examination by Ryan et al. [23], we could not identify an association between the impact region and distribution of specific injuries. In accordance with Demetriades et al. we found brain contusions and subarachnoideal hematomas being the most frequent head injuries [13].

Previous cadaver, dummy, and simulation studies demonstrated distinct trajectories of body movement and velocity at the time of impact [11,16,24]. Modern car designs, developed to attenuate the consequences of a pedestrian collision mainly stem from experimental kinematical studies. Under laboratory conditions and in larger vehicles, the initial head impact frequently occurs at the hood [11]. EuroNCAP test protocols still focus on hood impacts. Since 2005, these tests are mandatory for all automotive manufacturers in the European Union. For example, the sub-system-impactor-tests (developed by the European Enhanced Vehicle safety Committee (EEVC)) mimic the effect of a 40 km/h impact with a pedestrian moving laterally across the path of the car pushing head forms against the hood top. We noted that, under real life conditions, head impacts to the windshield occurred more often than impacts to the hood, are associated with higher collision speed, and higher injury severity. This is in accordance with previous results: in impacts >40 km/h, windscreen and A-pillar contact was responsible for 22% of AIS 3+ injuries (bumper 20%, bonnet 18%) [6]. Furthermore, an in-depth-analysis underlines the significance of vehicle type and impact speed on head impact location [11]. An analysis of 137 crash tests with impact velocities of 25-39 km/h showed that the head contacts the windscreen in most cases [16]. Another study reported 6/37 hood impacts but 10/37 windshield impacts amongst 37 pedestrian fatalities [21]. MacLaughlin et al. previously demonstrated a higher Head Injury Criterion in head impacts near the windshield compared to those in the middle of the hood [25]. In addition, casualties involving smaller cars (which are more frequent in Europe than in the U.S.) were found to be associated with higher head velocity and impact to the windshield [26].

It remains unclear whether the EuroNCAP impact speed of 40 km/h is an appropriate scenario, or if other speed levels should be tested additionally. Previous investigations reported that most serious injuries result from impacts >40 km/h [6] and with increased mortality at collision speed > 40 mph [2]. Our results indicate a comparable association. However, a previous report stated that the group aims at improving test methods and identifying additional impact scenarios [18].

Next to severe head trauma, blunt abdominal and chest trauma is a frequent life threatening condition after high energy impact [6,7,9,21,22]. The Australian fatal file reported that "head and chest injuries alone of AIS 4+ severity accounted for 20% and 16% of fatal injuries respectively and 39% in combination" [6]. Other autopsy studies found relevant abdominal injuries in 45% and 56% respectively [21,22]. Demetriades et al. presented specific organ injury rates showing that severe abdominal and chest trauma were significantly less frequent than severe head injuries, nevertheless posing a threat to life [13], requiring further attention, and standardized testing. Series that included lower-impact-velocities or excluded fatal cases did see significantly lower rates of abdominal trauma [9,10].

In our series, 11 spine fractures were documented, of which 9 where unstable. This is comparable with previous studies, which reported spine injuries being a substantial source of morbidity [6,13,27,28]. Lower limb injuries significantly contributed to the overall morbidity of the study sample. Our data suggest that the causation of leg injuries has not been fully understood. The causation of injury due to direct impact was described in several previous studies [12,16,17,21]. However, our data indicates that injuries to the contralateral leg are frequent and need further attention.

## Conclusions

Results from the IMPAIR study are in accordance with previous studies. Our data may assist in understanding the causation of injuries of pedestrians hit by cars. Our results indicate that the EuroNCAP protocols do not cover the entire range of head impacts and may not sufficiently predict effects observed under real life conditions. Further research should include life threatening injuries other than head injuries.

## Competing interests

The authors declare that they have no competing interests.

## Authors' contributions

US, MF and MB led the interpretation of findings while US was writing the manuscript. MB, MF, and JS performed on-site and clinical documentation, data entry and literature search. JS, DS, and AE led project design and steering. GM and DS participated with methods and statistical analysis. All authors reviewed the final draft of the manuscript.
